# Contextual Factors Influencing Cost and Quality Decisions in Health and Care: A Structured Evidence Review and Narrative Synthesis

**DOI:** 10.15171/ijhpm.2018.09

**Published:** 2018-02-28

**Authors:** Iestyn Williams, Hilary Brown, Paul Healy

**Affiliations:** ^1^Health Services Management Centre, University of Birmingham, Birmingham, UK.; ^2^NHS Confederation, London, UK.

**Keywords:** Healthcare Decision-Making, Cost, Quality, Literature Review, Health Management

## Abstract

**Background:** Decisions affecting cost and quality are taken across health and care but investigation of the mediating role of context in these is in its infancy. This paper presents a synthesis of the evidence on the contextual factors that influence ‘decisions of value’ – defined as those characterised by having a significant and demonstrable impact on both quality and resources – in health and care. The review considers the full range of resource/quality decisions and synthesises knowledge on the contextual drivers of these.

**Methods:** The method involved structured evidence review and narrative synthesis. Literature was identified through searches of electronic databases (HMIC, Medline, Embase, CINAHL, NHS Evidence, Cochrane, Web of Knowledge, ABI Inform/Proquest), journal and bibliography hand-searching and snowball searching using citation analysis. Structured data extraction was performed drawing out descriptive information and content against review aims and questions. Data synthesis followed a thematic approach in accordance with the varied nature of the retrieved literature.

**Results:** Twenty-one literature items reporting 14 research studies and seven literature reviews met the inclusion criteria. The review shows that in health and care contexts, research into decisions of value in health and care is in its infancy and contains wide variation in approach and remit. The evidence is drawn from a range of service and country settings and this reduces generalisability or transferability of findings. An area of relative strength in the published evidence is inquiry into factors influencing coverage and commissioning decisions in health care systems. Allocative decisions have therefore been more consistently researched than technical decisions. We use Pettigrew’s (1985) distinction between inner and outer context to structure analysis of the range of factors reported as being influential. These include: evidence/information, organisational culture and governance regimes, and; economic and political conditions.

**Conclusion:** Decisions of value in health and care are subject to range of intersecting influences that often lead to a departure from narrow notions of rational decision-making. Future research should pay greater attention to the relatively under-explored area of technical, as opposed to allocative, decision-making.

## Introduction

Many governments now find themselves faced with unprecedented constraints on their health and care spending capacity whilst demands and expectations continue to increase. This has led to the championing of investment and disinvestment decision-making that incorporates opportunity cost and budget impact, alongside quality and outcomes.^1^ The development and spread of formal coverage decision-making bodies internationally has prompted inquiry into the drivers of resource allocation decisions of this kind. However, significant investment decisions are also made in other areas: for example service redesign, and changes to workforce and governance arrangements. Although considerations of both benefit and resource impact arguably *should* infuse such policy and programme decision-making, this implies a level of rationality on the part of decision-makers which may not always be present, possible or even desirable in practice. Whilst the psychology of decision-making has been subject to much study and theorisation, such decisions are also likely to be influenced by aspects of *context*. This paper presents findings from an evidence synthesis carried out in order to understand the contextual factors that are influential in these decision-making domains, and which therefore facilitate or attenuate the pursuit of quality and affordability. The focus is on ‘decisions of value’ – defined as being characterised by a significant and demonstrable impact on both quality and resources. The paper begins with a definition of terms and an explanation of the scope and conceptual foundation of the review. This is followed by a description of the study objectives, methods and a comparative thematic analysis of findings. Pettigrew’s^2^ distinction between inner and outer context is used to structure analysis of the factors identified, and the interplay between them as influencers of decision-making. Results of the analysis are presented and discussed alongside recommendations for future theoretical and empirical enquiry, as well as for decision-making in practice.

### Decisions of Value in Health and Care

The term ‘decisions of value’ is used here to refer to decisions with substantial and direct implications for both cost/finance and quality/outcomes in health and care settings.^3^ Across health care systems there are powerful pressures on local decision-makers to improve outcomes whilst reducing expenditure.^4^ However, achieving these twin aims can be impeded by, for example, organisational siloes,^5^ and clinical-managerial division.^6^ In this study, we examine formal decision-making processes undertaken by, for example: governing bodies within health and care organisations; local government departments; healthcare insurance agencies; service planners, hospital senior management and so on. The focus on formal decision bodies means that continuous and/or covert decision-making, whilst important, is beyond our remit.^7^ Similarly, our focus is specifically on *meso* level decision-making tiers which include those at the organisational or inter-organisational level. Although the characteristics of such decision-making contexts will vary from country to country, in each case they are distinct from *macro* (eg, national/governmental) or *micro* (eg, clinical/practice) levels, each of which warrant separate study in their own right. These other decision-making tiers are therefore only included here to the extent that they, in themselves, constitute contextual factors influencing meso-level decision-making.

We take ‘decision-making’ to mean the act of selecting a course of action from among alternatives (including ‘do nothing’). Our focus is therefore on *option selection* rather than other decision features such as agenda setting, implementation and review.^8^ It is this aspect of decision-making for which the imperative to draw on best evidence to maximise outcomes is most often invoked.^9^ The logic of this rationality can be *allocative* (ie, relating to distribution of resources between alternative interventions or programmes) or *technical* (ie, relating to investments made in order to enhance organisational capacity and functioning). In this context we might consider allocative decisions to include for example: selecting treatments for inclusion in insurance packages or formularies, and purchasing or contracting for specific health and care services. Technical decisions might include: organisational mergers and takeovers; investment in programmes of service improvement or engagement; major workforce reorganisation; adoption of new technologies, organisational systems, and so on. This distinction is important as it is rare for the full range of decisions to be included in studies of decision-making (as we demonstrate in this paper) (Box 1).

**Box 1. Examples of Decisions of Value in Health and Care**

** Allocative:**

A local government agency commissions a service from the charity/third sector.

A health or care provider decides to invest in a new treatment, device or equipment.

A prescribing group decides to replace a treatment and thereby remove it from a formulary list.

**Technical:**

Two health and/or social care organisations decide to partially or fully merge, forming a new organisation.

A service planning body decides to downgrade or close an in-house service or organisation.

A provider organisation decides to undertake substantive internal audit, governance and/or review of its operations.

A provider organisation decides to adopt a set of new managerial structures and/or arrangements.

A provider organisation decides to invest in a major update of its physical or technological infrastructure.

A provider organisation decides to significantly increase or decrease its workforce levels.

A service planning body or provider organisation decides to lead a programme of funded service improvement.

A service planning body or provider organisation decides to invest in a programme of patient/public/stakeholder engagement.

There is a rich and longstanding theoretical literature which considers the rationality of decision-making and attendant requirements of perfect knowledge and predictability of decision outcomes.^10,11^ In particular, theories have centred on psychology and the mediating role played by cognitive biases and group dynamics such as consensus building and argumentation, as well as the influence of expertise and seniority.^12-14^ Characteristics of decisions and those charged with making them vary and have been found to be important in shaping decision outcomes.^15-17^ These characteristics include the complexity of the decision and extent of decision precedent, which influence both speed of decision-making and level of supporting information typically accessed in the decision-making process. Decision-maker characteristics such as professional role and values, personality, cognitive style and demographic factors such as age, length of tenure and education have been found to influence aspects of decision-making such as levels of risk-taking, volume and type of information sought.^18,19^

By comparison, investigation of the mediating role of context in decision-making is under-developed. Dobrow et al^15^ note that a ‘normative evidence-based’ mind-set is often somewhat at odds with a ‘practical-operational’ orientation, in which contextual factors are acknowledged as attenuating the strict application of best evidence. Contextual factors are to some extent accounted for in institutional approaches. These schools also question the explanatory power of instrumentalist models of decision-making, instead emphasizing the institutional outcomes of legitimacy and recognition, and counter logics of organisational isomorphism.^20^ In order to disaggregate the relevant features of this institutional context it is helpful to draw on Pettigrew’s^2^ broad distinction between inner and outer context:

*“Inner context refers to factors from within the organization eg, structure, culture, power and political characteristics; and outer, to factors external to the organization such as industry sector, economic, political and social context. This is a handy simplification, although may not be so easy to identify in practice, as these boundaries are sometimes permeable.”* ^21^

Frameworks such as that of Bate et al^22^ add to the category of inner context factors such as size, scale and complexity of the organisational unit; degree of organisational stability, and; prior financial and service performance. To the *outer* context they add factors such as: regulatory environment and market forces. However, settling on a definitive and granular categorisation is problematic given that, as Squires et al^23^ note ‘no one framework is sufficiently inclusive or comprehensive about what comprises context.’ What’s more, such frameworks have typically been designed to analyse change processes and it is not clear that the extent to which any explanatory power in this domain is transferable to the analysis of decision-making.

In this review we have grouped factors under descriptive headings selected to enable capture of all contextual factors reported in the included studies (see Box 2).

**Box 2. Categories of Factors**

** Sources of information:** refers to factors reported in the literature such as formal evidence and tacit information.

**Interests:** refers to the range of stakeholders that may seek to influence decisions, including professional, commercial, patients and so on. ‘Interests’ can be located predominantly in either the inner or outer context.

**Organisational characteristics:** covers factors such as size, structure and resource levels of the organisation in which the decision-making function is embedded.

**Governance and leadership:** refers to the modes of practice in relation to leading and managing the organisations within which the decision-making function is embedded.

**Geography:** covers factors such as extent of rurality and accessibility for patient populations.

**Economics:** refers to extent of available resources, and system payment mechanisms.

**Relationship to government:** refers to factors deriving specifically from political overseers and their agents, including regulation, contracts, services frameworks and standards.

Although we might assume that ‘dynamic decision-making’^7^ is the product of the interaction between such factors and human dimensions, the nature of these factors and this interaction with formal decision functions is not well understood in health and care settings.

In summary then, decisions of value are understood to be non-routine decisions that impact substantially and explicitly on both costs and outcomes, and which require consideration of options. The aims of this evidence synthesis are to understand the contextual factors that influence decisions of value in health and care, and to draw conclusions and identify areas for future enquiry. The specific objective is to identify and synthesise previous empirical studies of the relationship between contextual (inner and outer) factors and decisions of value.

## Materials and Methods

The method employed for this study is structured evidence review and narrative synthesis. Following initial scoping searches of online search engines (Google Scholar and NHS Evidence) a list of search terms and inclusion criteria were developed. Full searches were then carried out of health and social care databases keywords and abstracts (HMIC, Medline, Embase, CINAHL, NHS Evidence, Cochrane) and selected non-health databases (Web of Knowledge and ABI Inform/Proquest). Follow-up searches focussing on journals and mesh terms identified as most relevant from these initial searches were conducted along with hand-searching of identified bibliographies and reference lists. Snowball searching using citation analysis and bibliography scanning was then performed with a final google scholar search carried out in February 2015 (Box 3).

**Box 3. Example Search**

** Search strategy:** influences on cost/quality decision-making in health

**Databases:** CINAHL

**Search terms:** ‘Decision making’ or ‘Investment’ or ‘Management’ or ‘Governance’ or ‘Adoption’ or ‘Choice’ or ‘Selection’ or ‘Strategy’ or ‘Planning’ or ‘Quality’ or ‘Service improvement’ or ‘Improvement’ or ‘Innovation’ or ‘Cutbacks’ or ‘Rationing’ And ‘Causes’ or ‘Drivers’ or ‘Influences’ or ‘Factors’ or ‘Finance’ or ‘Cost’ or ‘Cost effectiveness’ or ‘Evidence’ or ‘Context’

Included documents were empirical (either new research or evidence synthesis) and published between January 1990 and February 2015 in academic peer reviewed formats. The review was international in scope but confined to English language reporting. Included items relate to formal and explicit decisions where options/alternatives are available (eg, ‘next best course of action’ or ‘do nothing’) with demonstrable implications for quality and finance, and which considered the influence of a contextual factor or factors on these, in a health and/or care context (Figure).

**Figure:**
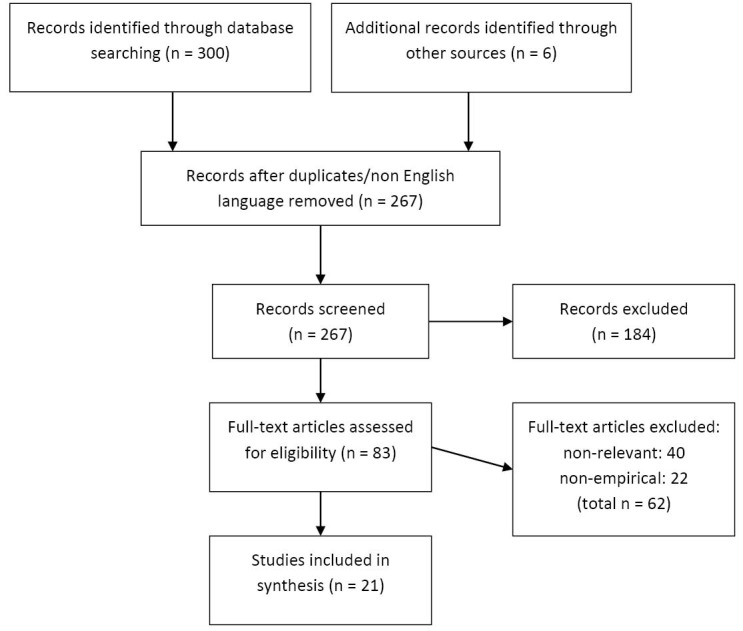
PRISMA Flowchart.

For all included items, structured data extraction was performed drawing out descriptive information and content against the review aims and questions (see Table 1). Data synthesis was conducted in accordance with the nature of the evidence base and a narrative, thematic approach was adopted as the best approach for combining studies employing divergent methods.^24^ Comparisons were made across studies in order to provide an overview of the main themes and characteristics of the evidence base against research aims and questions.^25^ A key aim was to identify factors reported as influencing decisions of value. A coding framework was developed from a combination of the self-reported categories employed in studies and additional categorisation work of the authors (see Table 2). This was applied and developed iteratively until each reported factor was assigned a code.

**Table 1:** Included Literatur

**Source**	**Decisions**	**Methods**	**Relevant Research Aims/Question**	**Relevance to Review**
Abelson^31^	Community decision-making processes in Canada	Case studies involving interviews, secondary sources and observation	Explores the role that context plays in shaping community decision-making processes	Covers allocative and technical decisions
Bazzoli et al^32^	Investment in plant and equipment in US hospitals	Quantitative analysis of routine data: on hospital finances, performance etc	To examine effects of financial pressure on hospital operations including investments in plant and equipment	Covers technical decision-making in a specific system setting
Castro et al^33^	Decisions over the adoption/diffusion of new innovations in Italy	Analysis of routine data, using regression analysis, on expensive medical equipment (eg, MRI), comparing public and private hospitals	To investigate the relationship between reimbursement systems and decisions to adopt technological medical innovations	Covers technical decision-making in a specific system setting
Denis et al^34^	Merger decisions in healthcare in Quebec, Canada	Longitudinal case study using documentary analysis and interviews	To analyse the determinants of a merger between two publicly funded hospitals	Covers technical decisions in a specific system setting
Dranove et al^35^	HMO formulary inclusion decisions, US	Survey of HMO directors of pharmacy analysed using logistical regression analysis	To identify economic and organisational factors that affect likelihood of inclusion of new drugs	Covers allocative decision-making in a US setting
Eddama and Coast^26^	Review and synthesis of the international literature on use of economic evidence in decisions to invest in healthcare interventions	Literature review	To investigates the role of economic evidence in healthcare coverage decision-making	Reviews allocative decisions on technology coverage across a range of settings
Fischer^36^	Review and synthesis of the international literature on allocative decision-making at the pan-organisational level	Literature review and documentary analysis	To summarise factors that influence decision outcomes and appraisal criteria as measured in quantitative studies	Reviews allocative decisions on technology coverage across a range of settings, excludes qualitative studies
Fraser and Estabrooks^28^	Review and synthesis of the international literature on home care decision-making	Literature review	To understand what factors influence case managers’ resource allocation decisions in home care	Synthesises literature on allocative decision although relatively little research identified
Fraser et al^37^	Case management resource allocation decisions in Canada	Ethnographic study of a home care programme using interviews, card sorts and participant observation	To explore factors that influence case managers’ resource allocation decisions in pediatric home care	Covers allocative decisions in a specific system setting
Hensher and Fulop^38^	Health authorities in London, UK	Survey and interviews	To assess the influence needs assessment has had on decision-making	Covers allocative and technical decision-making in a specific system setting
Kisa et al^39^	Financial decision-makers at organisational level in hospitals (public and private) in Ankara, Turkey	Survey of people in charge of financial decisions in 14 private hospitals and 66 outpatient clinics and imaging centres	To investigate how involved finance officers are in decision-making in healthcare organisations	Covers technical decision-making in a specific system setting
Li and Benton^40^	Capacity management decision-making (eg, expanding services, partnering, investing in technology, workforce management) in US hospitals	Questionnaire on hospital capacity management decisions and Practices, analysed using structural equation modelling	To measure influence of hospital size, location, teaching involvement, and service mix on hospital capacity resource management decisions	Covers technical decision-making in a specific system setting
Li and Benton^41^	Technology and nurse management decisions in US Hospitals	Questionnaire on technology and nurse management decisions analysed using structural equation modelling	To measure influence of hospital size and location on technology and nurse management decisions	Covers technical decision-making in a specific system setting
Miller et al^30^	UK Local government commissioning (ie, funding) decisions in the field of public health	Interviews with local government commissioners	To identify the information that influences decisions on public health spending	Covers allocative decision-making in a specific system setting
Paudyal et al^42^	Review and synthesis of the international literature on community pharmacist decisions to adopt new treatments	Literature review	To identify factors associated with community pharmacists’ adoption decision-making	Synthesises literature on allocative decision although relatively little research identified
Polisena et al^43^	Review and synthesis of the international literature on disinvestment in healthcare	Literature review	To review the application of frameworks and tools for disinvestment decision-making in health and social care	Covers allocative and technical decisions across range of settings
Roggenkamp et al^44^	US hospital decisions regarding adoption of case management	Routine data analysis	To investigate the adoption of case management by US hospitals at three-time periods:1994, 1997, and 2000	Covers allocative and technical decision-making in a specific system setting
Sosnowy et al^45^	State level health leaders in the US state of New York	Mixed qualitative methods including individual and group interviews	To determine the use of decision-making processes by state local health department leaders and barriers/facilitators to use of evidence-based decision-making	Covers decision-making in a specific system setting. Precise nature of decisions is somewhat unclear
Vuorenkoski et al^29^	Review and synthesis of the international literature on macro/meso level coverage decision-making in industrialised countries	Literature review	To analyse coverage decision-making processes	Covers allocative and technical decisions across range of settings. Not designed specifically to measure factors influencing decisions
Williams et al^27^	Health coverage decision-making in England and elsewhere	Literature review followed by documentary analysis and case studies (interviews and observation)	To investigates the role of economic evaluation in healthcare decision-making	Covers allocative decisions across range of settings
Wright and Martin^46^	Community health centre decisions in the United States	Qualitative interviews	To explore the role of consumer trustees in decision-making under economic constraint	Covers allocative and technical decision-making, focussing on one specific contextual influencer

**Table 2:** Summary of Contextual Factors Cited by Literature Item

**Decision Type**		**Author/Research Tradition**	**Contextual Influencers Identified**
Technical decisions at the organisational and sub-organisational level	Investment in hospital infrastructure and operations	Bazzoli et al^32^/Health services research	Financial pressures Economic climate
	Decisions to adopt innovations	Castro et al^33^/Health services research	Budgetary constraints Market (demand and supply) forces Complexity/case mix External competition Payment system
	Decisions to merge organisations	Denis et al^34^/Political science	Internal power relations External political context
	Finance decisions in hospitals and clinics	Kisa et al^39^/Health services research	Finance officers Market forces Financial pressures
	Capacity management decisions (expanding, partnering, investing, workforce management etc)	Li and Benton^40^/Operations research	Hospital size and location, Hospital status (eg, teaching) Service mix
	Technology and nurse management	Li and Benton^41^/Operations research	Hospital size Hospital location Technology investment
Allocative decisions at the (sub)organisational level	Health coverage decisions	Dranove et al^39^/Health services research	Relationship with drug manufacturers Profit/non-profit status
		Eddama and Coast^26^ (review)/Health services research	Organisational/institutional constraints External and internal politics Cultural characteristics of the organisation
		Paudyal^42^ (review)/Health services research	Patient safety information Endorsement by medical bodies Country characteristics (variation across settings)
		Vuorenkoski et al^29^/Health services research	Clinical evidence Costs of treatment information Social value considerations
		Williams et al^27^/Health services research	Clinical effectiveness information Cost effectiveness information Organisational/institutional constraints
		Polisena et al^42^/Health services research	Disease burden information Clinical effect and patient safety information Costs and cost effectiveness information Health service impact (ethical, legal, and psychosocial) information
	Adoption of case management	Roggenkamp et al^44^/Healthcare management	Institutional forces Economic incentives
Allocative decisions at the super-organisational level	Health coverage decisions	Eddama and Coast^26^ (review)/Health services research	Organisational/institutional forces
		Fischer^36^/Health services research	Clinical information Economic information Ethical considerations
		Vuorenkoskiet al^29^ (review)/Health services research	Cost information Past decisions Severity of disease information Patient demand Clinical opinion Pharmaceutical company behaviour
		Williams et al^27^/Health Services Research	Clinical effectiveness information Cost effectiveness information Organisational/institutional constraints
	Resource allocation in home care	Fraser and Estabrooks^28^/Health services research	Client characteristics Policy constraints System constraints (work load and volume, staff turnover, organisational structure) Resources
		Fraser et al^37^/Health services research
	Investing in preventive/public programmes	Miller et al^30^/Public management	Political context Interests
	Disinvestment decisions	Polisena et al^43^/Health services research	Disease burden information Clinical effect and patient safety information Costs and cost effectiveness information Health service impact (ethical, legal and psychosocial)
	Health planning decisions	Hensher and Fulop^38^/Health services research	Needs assessment Political bargaining between interest groups

The interdisciplinary nature of the evidence base and the challenges of applying quality criteria across research paradigms meant that assessment of each included item was confined to considerations of relevance rather than research quality (see Table 1). However, by excluding non-peer-reviewed literature we ensured only studies with explicit methods and which followed a defined research designs were included. Where previous evidence syntheses of sub-sections of the literature were identified which meet the inclusion criteria, we incorporated the prior synthesis to our own instead of disaggregating and re-analysing each of the relevant studies contained within them. For example, Eddama and Coast^26^ and Williams et al^27^ both review the literature on the influence of economic information in allocative decision-making and we have reviewed and incorporated their analysis and conclusions.

## Results

Twenty-one literature items reporting 14 research studies and seven literature reviews met the inclusion criteria. Six of the research studies were carried out in the US with three from each of the United Kingdom and Canada and the remaining two from countries in Europe and Asia. Of the empirical items included, eight reported from research into allocative decision-making, five reported on research into technical decision-making and six of these covered both. Four reviews covered allocative decision-making and two covered both allocative and technical decisions. Details of the included studies are presented in Table 1 and a breakdown of the factors reported in each included literature item are presented in Table 2.

### Strengths and Limitations of the Evidence

The review shows that in health and care contexts, research into decisions of value is in its infancy and contains wide variation in approach and remit. For example some studies seek to identify inductively the full range of influencing factors whereas others measure correlations between a narrower range of pre-identified factors and a dependent variable. This prevents us from aggregating the reported influence of factors across studies. Combined with the lack of replication or critical appraisal of studies – especially in relation to technical decision-making – this makes it premature to issue definitive statements regarding the *relative* influence of factors.

As well as this, the evidence is drawn from a range of service and country settings – albeit our searches identified few studies from lower and middle-income countries – and this reduces generalisability or transferability of findings. Furthermore, the variety of definitions for phenomena such as ‘leadership,’ ‘culture’ and ‘resources,’ means that assessment of their power as influencers is subject to uncertainty. These variations reflect differences of research tradition. For example although the literature is dominated by health services research it contains contributions from management studies, operations research and political science, and draws from both qualitative and quantitative research paradigms. More work is therefore required to develop a taxonomy of factors that can be clearly defined, measured and analysed in different settings and to help facilitate reconciliation of insights from these divergent schools.

An area of relative strength in the published evidence is enquiry into the factors influencing coverage and commissioning decisions in healthcare systems.^27-30^ The factors influencing these allocative decisions have therefore been more consistently explored than factors affecting technical decision. The greater variety in *technical* decision-making makes it difficult to draw definitive conclusions regarding the influence of contextual factors. These caveats notwithstanding, the following sections describe inner and outer contextual factors and their influence as reported in the literature.

### Inner Context

Factors deriving from the inner context reported as influential include information accessed by decision-makers; interest groups within the organisation; organisational characteristics and governance structures.

### Sources of Information

Levels of information and analytical resources are reported as important in shaping decisions of value, especially in relation to allocative decision-making. For example, technology coverage decisions have been found to be influenced by clinical, ethical and cost information.^29,36,43^ *Absence* of such information is also reported as important: for example high levels of uncertainty in the face of information deficits have been shown to reduce adherence to a instrumentalist decision-making model and to open up determinations to greater levels of judgement and intuition.^27^ Despite these findings, the relative importance of information (or its absence) can be over-stated and may be skewed by the prevalence of its pre-selection as a variable for analysis.^26,27,30^ Importantly, even in these studies information is invariably found to vie for primacy with other contextual drivers and influences.^28,30,38^

The role of information in technical decision-making at the organisational level is less well understood. Such evidence as exists suggests that decision-makers consult a range of information sources incorporating both explicit and tacit knowledge.^30,45^ These sources include professional journals, legal advisors, the media and the experiential information provided by other decision-makers, as well as advice from specialists. The relative importance attached to each source varies according to decision-maker characteristics such as age, occupation and education levels, as well the nature of the decisions themselves. For example highly technical areas of decision-making typically engender greater reliance on specialist information and advice. Professional roles appear to mediate the importance given to information: the literature contains instances of differences between decision-makers’ emphasis on quality and cost considerations, with clinicians more likely to emphasize the former and budget holders/finance professionals emphasizing the latter.^27,33^

The extent to which an organisation is able to identify and process new knowledge is likely to affect levels of rationality (ie, instrumentalism) in decision-making. However, as noted above, this knowledge is not confined to formal evidence. The literature provides support for the importance of tacit knowledge located in organisational memory and therefore of decision-making antecedents. However, workload levels are an important mediating variable in this regard and budgetary deficits have been cited as militating against an evidence based decision-making approach.^45^

### Interests

The underlying premise of much of the discussion of interests is a concern with how power and self-interest are enacted by those not directly involved in the decision-making process. In general, internal actors and interests are reported as being highly influential in decision-making.^38,34^ However this influence can be uneven with, for example, ‘experts’ found to be more influential than lay or patient stakeholders in priority setting.^29^ Wright and Martin^46^ conclude that ‘consumer governors’ in US community health centres are less influential than other stakeholders (eg, clinicians) even in relation to functions such as identification of community needs. Williams et al^27^ explore how interests are advanced through mobilisation of factors such as evidence and expertise, indicating the interrelationship between multiple factors within the inner context.

### Organisational and Institutional Characteristics

Technical decision-making in particular is subject to the influence of organisational characteristics such as size, financial performance and service mix. In relation to size and service mix, Li and Benton^40^ conclude from a US survey that:

*“Larger hospitals are more interested in expanding outpatient services, forging partnerships with physicians and managed care delivery systems, and seeking effective demand management decisions.*”

Service mix is also influential in technology adoption decision-making.^40^ For example teaching hospitals typically have more specialised and complex medical services, thereby increasing the resources and expertise available to them to support adoption decisions. The availability of slack resources for decision support and implementation, which are linked to organisational size, can affect decisions affecting costs and quality.^28,47^ However, the relationship between financial conditions and decision-making is complex and often unpredictable. Budgetary deficits have been found to militate against an evidence based decision-making approach.^45^ What’s more, the uneven distribution of resources within and between organisations can lead to disparities of influence between interest groups.^34^ This again highlights the interrelationship between inner contextual factors such as resources, interests and organisational structure.

In some studies the term ‘institution’ is used to refer to characteristics of the broader (ie, supra-organisational) sector within which the decision-making function is located. Roggenkamp et al^44^ conclude that the foremost influences on decisions to adopt hospital case management are institutional rather than economic. By way of illustration they note that those most likely to benefit economically are not necessarily the most likely to adopt. Instead, they find inter-organisational factors such as the behaviour of competitors to be a more important predictor of decision-making. The literature includes multiple other references to institutional influence but with little commonality of meaning. For example, the term is employed as a synonym for organisations in some studies, and for market factors in others. Much of the detail of institutional influence is therefore discussed here under different headings.

### Governance and Leadership

Extent of centralisation and specialisation has been linked to organisational performance, although less is known specifically about the impact of these on decision-making. In general there is a normative strain in the literature advocating decentralisation of decision-making and flatter management structures with increased autonomy at the front line.^48^ This links to the claim that autonomy and discretion/responsibility are important in enabling rational decision-making. Respondents in Sosnowy and colleagues’^45^ study cite the importance of ‘evidence-based’ decision-making being promoted and supported by the leadership of the organisation. However more research is required into how these factors and others such as reporting relationships affect decisions of value.^35^

### Organisational Culture

Although Eddama and Coast^26^ identify culture as a significant variable affecting the extent to which ‘rational,’ evidence-based decisions are made on investment in health and care, overall the review also notes that organizational culture and strategic orientation are not well understood in relation to decision-making. There has been extensive research into the values and norms that predominate in healthcare organisations^49,50^ and although there is a growing literature on the relationship between culture and performance there is little that focusses on decision-making either as an endpoint or an intervening variable. Indeed ‘culture’ has been described as the hardest organisational concept to define and this makes it difficult to measure its impact on decision-making.^51^ Clearly we might infer that culture shapes decision-making but there remains little by way of an evidence base on how this happens.

### Outer Context

Influential factors deriving from the outer context include: geographical location; payment and reimbursement regimes; economic climate and; government and regulatory factors.

### Geography

Geographical location has been found to be influential in relation to technical decision-making. For example decisions taken by health and care providers in rural areas are likely to be different to those taken in urban areas for reasons which include the skills requirements and capabilities of the workforce and the profile of patient populations. Li and Benton^40,41^ identify a greater emphasis on workforce development in rural areas where recruitment is often more constrained. Location therefore affects staffing decisions but can also be linked to factors such as case mix and complexity. This illustrates the interrelationship between inner and outer contextual factors, especially as traversed by professional networks which can be both within and outside of the decision-making organisation.^29,42^

### Interests

A variety of groups external to the decision-making organisation can and often do exercise influence. These include members of the public, the media, legal bodies and professional representative bodies. The role that such parties play in allocative decision-making processes is better understood than it is in technical decision-making in health and care contexts.^26^ The media is frequently invoked as a counterforce to rational decision-making in its apparent promotion of unrealistic expectations and sensationalist causes, and/or in its role as a mouth piece for dissatisfied stakeholders.

### Economic Factors

Economic factors in the form of resource pressures have consistently been found to influence technical decision-making at the organisation level. For example Bazzoli et al^32^ found that financial constraints contributed to decisions to reduce healthcare investment, and Roggenkamp et al^44^ found economic factors to be influential in decisions to adopt a case management approach in US hospitals. It is perhaps axiomatic to allocative decision-making that economic considerations are taken into account, although in practice these are often found to be secondary to other considerations.^26,27^

The influence of payment systems is illustrated in the literature through studies of, for example, the effects of reimbursement mechanisms on technology adoption. Castro et al^33^ found that a payment-per-case reimbursement system to be correlated with reduced rates of innovation adoption decisions, and elsewhere system characteristics have been found to influence case managers’ resource allocation decisions.^28^ Similarly, Dranove et al.^35^ found that non-profit status made inclusion of new drugs on healthcare formularies more likely.

### Relationship to Government

The role that government and/or regulatory bodies play in decision-making has been emphasized in a number of fields and this can affect organisations or individual decision-makers operating within them.^45,37^ For example, hospital merger decisions have been found to be influenced by government pressure especially where public resources are the only funding source.^34^ Overall, much of the literature included within the review did not directly report on factors such as regulation, government contracts, service frameworks and standards.

### Intersecting Factors

To mitigate factor selection bias in included literature, this table excludes studies where only a single influencing factor was selected for analysis (eg, Wright and Martin^46^). The literature clearly indicates that whilst factors can be disaggregated for analytical purposes they should not be treated as independent and many studies demonstrate how they intersect. For example, contextual factors are shown to affect levels of public engagement in decision-making,^31^ and hospital pharmacist drug adoption decisions are found to be influenced by a plethora of factors including: attributes of the medicine, professional opinion, resources and expertise, ethics and values, and patient opinion.^42^ Similarly, case manager resource allocation decisions are found to be shaped by a combination of system-related, home care program-related, family-related, client-related factors,^37^ and evidence and interests are often intertwined in shaping decision outcomes.^27^ Dependent variables are themselves shown to act as factors influencing subsequent decisions. For example high levels of hospital investment in technology have been found to lead to high levels of investment in nurse training.^41^

## Discussion and Conclusions

Enquiry into the relationship between quality and cost considerations in health and care decision-making is hampered by definitional confusion and there has been relatively little systematic exploration based on a shared conceptual understanding. Evidence synthesis therefore requires negotiation of the different terminologies that characterise the various literatures (as illustrated by the confusion noted earlier over the term ‘institution’). The disciplinary variety encompassed in our included literature, and the attendant divergence in theoretical and methodological approaches, places serious caveats on the analytical claims that can be made. It is clear that study findings are heavily shaped by their design and by the contours of the research traditions from which they derive. In particular these limitations make it difficult to draw inferences about the *relative* importance of contextual factors in health and care decisions of value.^52^ It is also important to note that our sample of literature is heavily skewed towards high income countries, with only one middle income country study^39^ and none from lower income countries. However there are a number of observations that can reasonably be made with regard to the interplay of inner and outer context in shaping decisions of value in health and care. In this section of the paper we consider the conclusions that can be drawn based on the evidence presented thus far, and identify implications for theory, research and practice in relation to decisions of value.

Decision-makers do not operate in a vacuum and there are strong clinical, financial, and political imperatives that constrain choices. Within the inner context these are most pronounced in relation to technical rather than allocative decisions, and yet these decisions are less frequently investigated in the literature. Our analysis implies that technical organisational decision-making is more directly circumscribed by prevailing structures of incentives, penalties and rewards as well as the dominant organisational culture and relationships. By contrast allocative decision-makers are often granted partial separation or autonomy, and perhaps as a result are more often considered to exemplify an instrumentalist model of evidence-based and rational decision-making.

The review suggests that outer-contextual factors also play an important role in shaping both allocative and technical decisions of value. In other settings it has been found that degree of external control is inversely related to the degree of rationality adopted in decision-making^18^ and that environmental factors such as hostility and/or munificence in the political environment can be highly influential.^19^ In governmental health and care systems the sheer volume of external oversight and regulation mechanisms, not to mention legal opinion and precedent, can engender decision-making driven by compliance and risk aversion rather than outcomes. Hostile contexts can induce stress which in turn has been shown to influence decision-making.^53,54^ and similar claims have been made for external factors which increase levels of decision risk and uncertainty.^55^ In these situations, decision-makers are more likely to fall back on intuition and experience than rational calculation.^56^ The implications of our analysis are therefore that excessive reform, regulation and scrutiny can induce response mode or risk-averse behaviour.

The nature of influence can be complex and multi-faceted, and the more distant the environmental factors the more difficult influence is to infer. There is a growing realisation that not only are the goals and values of much decision-making ‘fuzzy’ but the environment in which decisions are taken are also similarly fuzzy.^57^ The literature on complexity in health and care systems suggests that the relationship between decision-making and any single contextual factor is therefore unlikely to be linear. An ecological approach to understanding health and care systems would suggest that it is the multi-directional horizontal and vertical interplay between determinants and decision-makers that produce decisions and therefore the need to examine this interplay and its manifestations in specific settings.

Our review resonates with debates between normative rational choice theories of decision-making and descriptive organisational theories which emphasize context and environment.^57^ This is not the first time that decision-making has been shown to be complex and contingent on contextual factors. However, these empirical and theoretical insights are relatively under-explored in the health and care environment which remains heavily influenced by narrow, normative conceptions of decision-making which take insufficient account of the multiple and conflicting goals of governments and their agents at the meso level.^58^ A more responsive rationality, in which multiplicity is negotiated iteratively according to changes in context, is likely to be more practically useful.^59^

It is clear from the review that the variety and complexity that characterises decisions of value in health and care confounds simple prescriptions for improvements to practice especially considering mediating factors such as the nature of the decision (scale, levels of certainty, expected impact). Allocation of resources to, for example, service expansion and contraction, staff training, recruitment, public engagement and so on, will only be effective where it is informed by a detailed understanding of local context. Calculation of these factors as well as the expected controversy and impact of decisions could help determine the amount of time and information required to discharge decision-making as well as the extent to which prior buy-in will need to be secured from affected parties.

In relation to information, levels of resource mobilised should be roughly commensurate with the scale and likely impact of decisions. Rational decision-making is enhanced where investment in option appraisal, decision modelling, and other forms of information and analysis is greatest. However this should be offset against opportunity cost of investing resources in this area. A good example of this is formal cost-effectiveness analysis which has been applied with some success to allocative decision-making at a macro level but which remains something of an expensive luxury at sub-tiers.^60^ The implications of these insights for decision-making in health and care are that important factors to consider include whether sufficient investment is made in the resources required to generate and interpret information relevant to decisions, and whether both explicit and tacit knowledge channels are facilitated.

Finally, the review has underlined the influence of interest groups. Where decisions affecting costs and quality are of significant scale and scope there is a strong normative case for involving patients and citizens. The logic of involving the public relates to their voice in relation to how public resources are spent and therefore has particular salience in relation to allocative decisions – for example priority setting, commissioning and disinvestment. The logic of involving patients derives primarily from their status as the intended beneficiaries of health and care services and their expertise in relation to understanding quality.

Just as it has been argued that alignment between organisational operating mechanisms and decision mechanisms, facilitates better organisational decision-making,^61^ our review underlines the importance of alignment with wider context. This suggests the importance of investigating how the factors identified interact and cohere in local settings. To this end, there is a requirement for development of a conceptual schema combining influential factors related specifically to decision-making. We hope that this paper sensitises us to key concepts and terms to inform such work, and that in time it will help to facilitate comprehensive, multivariate factor analysis across a range of decisions.

## Acknowledgements

The authors acknowledge the NHS Confederation, London, UK and the Academy of Medical Royal Colleges for funding this literature review as part of a wider investigation into ‘decisions of value in healthcare.’

## Ethical issues

Not applicable.

## Competing interests

Authors declare that they have no competing interests.

## Authors’ contributions

IW led the process of search strategy design and contributed to analysis. He led write-up of the manuscript. HB carried out searches and contributed to analysis and write-up of the manuscript. PH contributed to search strategy design, analysis, and write-up of the manuscript.

## Authors’ affiliations

^1^Health Services Management Centre, University of Birmingham, Birmingham, UK. ^2^NHS Confederation, London, UK.
